# Study of Health-Related Quality of Life and Healthcare Utilization among Type 2 Diabetic Population in an Urban Area of Eastern Nepal

**DOI:** 10.1155/2020/8839905

**Published:** 2020-12-24

**Authors:** Sangita Shah, Nilambar Jha, Deepak Kumar Yadav, Prajjwal Pyakurel, Sanjib Kumar Sharma, Suman Bahadur Singh

**Affiliations:** ^1^School of Public Health and Community Medicine, B.P.Koirala Institute of Health Sciences, Dharan, Nepal; ^2^Department of Internal Medicine, B.P.Koirala Institute of Health Sciences, Dharan, Nepal

## Abstract

**Introduction:**

Diabetes mellitus is a major cause of morbidity and mortality and places huge burden on public health funding. Diabetes affects quality of life through associated complications, comorbidity, and disease burden. Consequently, people have frequent healthcare visits. This study assessed quality of life and healthcare utilization patterns among type 2 diabetic populations in an urban area of eastern Nepal.

**Methods:**

A cross-sectional study was conducted among 270 participants of age ≥20 years with type 2 diabetes in Itahari using a semistructured questionnaire. A D-39 questionnaire was used to assess quality of life. Five wards were selected by systematic random sampling, and the population was proportionate according to the sample size. Multiple linear regressions were conducted to identify the factors associated with quality of life and its domains.

**Results:**

The highest mean score ± SD was found in the domain anxiety and worry (57.34 ± 11.08). About 18.5% of the participants perceived extremely affected quality of life. Hypertension (55.55%) was the most common comorbidity. Age, marital status, literacy, alcohol, disease duration, comorbidity, and complications were significantly associated with overall quality of life. In last 6 months of duration, 93.7% had hospital visits. Among them, 8.1% had emergency visit and 5.9% were admitted in the hospital.

**Conclusion:**

People with diabetes in this study were more affected in the domain anxiety and worry. The frequency of healthcare access and utilization in patients with type 2 diabetes was high. The quality of life among them could be improved by taking care on healthy behavior, comorbid conditions, and complications.

## 1. Introduction

Due to social and economic transition, leading to behavioral and metabolic risk factors, diabetes is leading as an emerging major public health problem in Nepal, with rising prevalence and its complications, especially in urban populations [1, 2]. It is a chronic, incurable, costly, and largely preventable noncommunicable disease which is responsible for millions of deaths globally [3].

Diabetes has multidimensional effect in QOL such as social, physical, and role functioning, worries about the future, and emotional and general well-being [4]. For this, people living with diabetes require regular visits to several healthcare professionals and utilization of the service of clinics and outpatient centres [5, 6]. This study assist healthcare practitioners, institution, policy makers, and community people to better understand quality of life and healthcare utilization in type 2 diabetes mellitus [7].

This study is aimed to assess the health-related quality of life and healthcare utilization among type 2 diabetic population in an urban area of eastern Nepal.

## 2. Materials and Methods

A community-based cross-sectional study was conducted among 270 type 2 diabetes patients residing in Itahari, a submetropolitan city to assess their health-related quality of life and healthcare utilization pattern. The study population comprised of all the type 2 diabetes patients aged 20–80 years, diagnosed for more than 1 year of duration. Among the 26 wards of Itahari, 5 wards were selected by the systematic sampling method. The population in each ward was proportionate according to the sample size (270), and the final sample from each ward, i.e., 5, 8, 17, 16, and 23 was 133, 62, 29, 24, and 22, respectively. Patients were recruited through the nonprobability sampling method by taking help from the local health personnel such as Female Community Health Volunteer, Primary Healthcare Record, other private clinic/hospital, pharmacy, and other health agencies. After receiving informed consent, a face-to-face interview was conducted using pretested semistructured questionnaires and a standardized tool, the D-39 questionnaire. Permission was granted by the author to use this questionnaire. Privacy, confidentiality, and anonymity were assured and maintained.

All the collected data were entered in Microsoft Excel 2007, and the cleaned data were converted into Statistical Package for Social Sciences (SPSS) 11.5 version for statistical analysis. The descriptive data were expressed in terms of frequency, percentage, and mean with standard deviation along with graphical and tabular presentation of the data. The Student *t*-test and one-way ANOVA were applied to find out significant difference between health-related quality of life and other related variables at 95% confidence interval where *p* < 0.05. The variables that were significant at *p* < 0.1 from the bivariate analysis were considered for multivariate analysis using multiple linear regressions where the backward linear regression method was specified in order to find confounders and/or effect modifiers. The level of significance was *p* < 0.05 for all the tests.

## 3. Results and Discussion

### 3.1. Results

Table 1 shows the demographic characteristics of type 2 diabetic patients in the study. The mean age ± SD (range) of the respondents was 53.86 years ± 11.89(26–79).More than half (54.4%) of the participants were obese followed by overweight (22.2%).

Half of the participants (52.6%) were suffering from diabetes for less than 5 years followed by 5–10 years (33.7%). Many of them (57.4%) were having additional illness other than diabetes, and 18.9% were suffering from the complication of diabetes.

**Figure 1:**
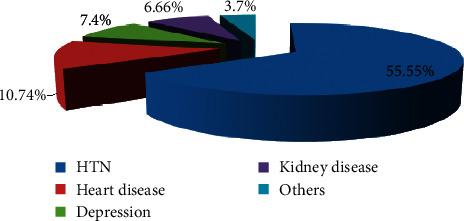
Prevalence of comorbidities among the participants; multiple response was provided by the participants with multiple diseases.

Out of the total participants, 55.5% had hypertension as the comorbidity (Figure 1).

**Figure 2:**
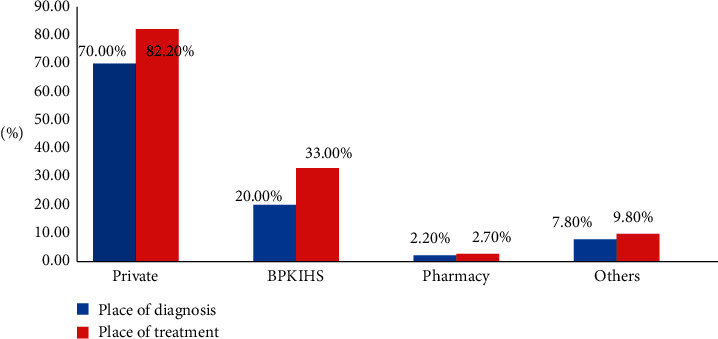
Distribution of participants according to place of diagnosis and treatment; multiple response was provided by the participants for visiting different health care facilities for diagnosis and treatment.

Most of the participants were diagnosed (70.0%) and treated (82.20%) at a private health centre followed by BPKIHS (Figure 2).

Table 2 presents the domain-specific QOL scores among the study participants. The highest mean score (SD) was found in the domain anxiety and worry (57.34 ± 11.08).

Table 3 indicates that the factors such as age, ethnicity, marital status, alcohol, education, occupation, blood pressure, diabetes duration, comorbidity, complications, family history, treatment, history of ER visit, and hospital admission in last 6 months were significantly associated with overall QOL. Age was found to be an important factor for quality of life in the diabetes patients.

The results of multiple linear regressions revealed that age, marital status, education, alcohol status, duration of disease, comorbidity, complication, and emergency visit were significantly associated with overall QOL of patients with diabetes (*p* < 0.05). These variables accounted for 40.0% of the variance of the total HRQOL. Age, alcohol status, comorbidity, complication, and ER visit were found significant in the four domains, energy and mobility, diabetes control, anxiety and worry, and social overload. In every domain, participants with age >50 years increased the mean score by more than 3 (Table 4).

### 3.2. Discussion

Going through various literature studies, to the best of our knowledge, this is the first community-based cross-sectional study in an urban setting of Nepal that assessed domain-wise impact in quality of life among diabetes patients and explored the factors significantly affected their QOL.

More than half of the participants (57.4%) in this study suffered from at least one comorbidity which agrees with the community-based cross-sectional study done in Khartoum, Sudan [8]. Hypertension was found to be the most common comorbidity (55.55%) in this study. Among those having comorbidity, 96.8% had hypertension. One of the studies done in a tertiary care diabetes centre in Karachi-Pakistan found that 57.2% of the participants were hypertensive [9]. This finding was also in agreement with the study done in polyclinic of Benghazi City [10]. This could conclude that diabetes and hypertension are closely related morbidities.

Treatment from private health sector was sought by 82.2% of diabetic patients. One of the studies done in Mumbai, India, among urban slums found that 81% of participants seek treatment from private health sector [11]. This could be linked with dissatisfaction of public hospital services and easy access to the private health centre.

As regular blood sugar examination is an important part of the treatment in diabetes patients, about 94% of the participants in this study visited the hospital for it. This act as a mirror in the finding of the study done among slums in Mumbai, where 75% of the participants visited healthcare in last 6 months [11]. The increase in awareness about the disease is the reason for higher percentage of participants visiting health centres, but the difference between both studies reveals that people of Itahari, submetropolitan city, are economically more strong.

The highest mean score (SD) was found in the domain anxiety and worry (57.34 ± 11.08).This reveals that most of the participants' QOL is affected in this domain. A cross-sectional study performed at two Basic Healthcare Units (BHU) in the western district of the City Health Department of Ribeirão Preto in 2012 [12] showed the highest mean score in social overload dimension and one of the hospital-based study in Kathmandu, Nepal [13]; quality of life was mostly affected in the domain physical health. These both findings are contradicted with the study finding. It can be predicted that although the disease is socially being acceptable, the patients are worried about their future health outcomes and economic burden.

After adjusting for the other variables, having age more than 50 years was associated with affected quality of life in the domain: energy and mobility (<0.001), diabetes control (<0.001), anxiety and worry (<0.001) and social overload (<0.001), and overall QOL by 5.460,4.249,4.237, 4.030, and 3.970, respectively. Similarly, an earlier study done in Riyadh, Saudi Arabia, found the group with over 50 years of age was significantly associated with lower quality of life on subscale physical functioning (*p* < 0.001) and role of emotional (*p* < 0.01) [14]. The study in US adults showed quality of life, in which domain physical functioning and social functioning were affected with increase in age, but in mental health, it is affected in the younger group [15]. The similar finding was found in the study done among Dutch patients, where older patients reported lower quality of life than the younger one [16]. This signifies as the people become old, physical activity is low, associated with different comorbidities and complications and worried about their health-related outcomes.

Significant association was found with increase in the duration of disease in the domain: energy and mobility (*p*=0.007) and overall QOL (0.039). The quality of life was affected by 2.686 and 1.845, respectively, in those who were suffering from the disease for more than 5 years. This was comparable with the study done in Saudi Arabia patients concluding that the longer duration of disease is associated with poor quality of life [14]. This was also consistent with the finding from the study done among females with type 2 diabetes referred to the Diabetes Clinic of Khoy City, Northwest of Iran [17]. This infers that the incidence of diabetes complications rises with the increase in the disease duration, which in turn, negatively affects the patients' QOL. However, also a contrary result was found in the study done in Kathmandu, Nepal, explaining having diagnosed for more than 10 years increased the quality of life in physical health domain by 5.184 score [13]. An observational study carried out in Dr. Sardjito Hospital in Yogyakarta, Indonesia, demonstrated no significant association between disease duration and the diabetes QOL clinical trial questionnaire [18].

The presence of comorbidity was found to be significantly associated with quality of life affected in the domain of energy and mobility (*p*=0.012), diabetes control (*p*=0.020), social overload (*p*=0.028), sexual behavior (*p*=0.001), and overall QOL (0.003) by 2.583, 2.334, 2.789, 5.649, and 2.807, respectively. This finding was similar with the finding of the study done in US adults, which showed that with the presence and the number of comorbidity, quality of life is affected in the domain of physical functioning, social functioning, and mental health [15]. The study done among females in Iran also found the consistent finding [17]. However, also a contrary result was found for the mental health domain which was not significant in the present study.

Those participants having an emergency room visit in last 6 months showed that the quality of life was affected in the domain: energy and mobility (*p*=0.005), diabetes control (*p*=0.007), anxiety and worry (*p*=0.027), social overload (*p*=0.006), and overall QOL (*p*=0.001) by 4.596, 4.364, 5.026, 5.644, and 4.948, respectively. The study done among US adults with diabetes indicated that limited access to healthcare especially low frequency or no use of healthcare services was associated with poor glycemic control, which may lead to poor quality of life [19]. This finding was contrary with the finding of the study done among US Shield surveys, where no association was found between ER visit and quality of life of the diabetes patients [5].

Those participants having history of hospital admission in last 6 months showed poor QOL in the domain, of energy and mobility, social overload, and overall QOL. This was comparable with the finding of a study done among US adults having diabetes and was a part of the marketing company using the SF-20 questionnaire (physical function, social function, and mental health) [15].

## 4. Conclusions

In this study, the quality of life of the participants was more affected in the domain of anxiety and worry with the highest mean score (SD) (57.34 ± 11.08).The utilization of private health facilities is high for both diagnosis (70%) and treatment (82.2%), which concludes that people in this region are more aware about their health outcomes.

Age, marital status, education, alcohol consumption status, duration of disease, comorbidity, presence of complications, and emergency room visits were significantly associated with overall QOL.

## Figures and Tables

**Table 1:** Demographic characteristics of the participants (*n* = 270).

Characteristics	Categories	Frequency	Percentage (%)
Age (years)	20‐40	37	13.7
	41‐60	159	58.9
	61‐80	74	27.4

Mean age ± SD (range) 53.86 years ± 11.89 (26‐79)			
Sex	Male	136	50.4
	Female	134	49.6

Marital status	Married	229	84.8
	Widow	31	11.5
	Unmarried	5	01.9
	Separated	5	01.9

Ethnicity	Brahmin/Chhetri	129	47.8
	Janajati	95	35.2
	Muslim	19	07.0
	Madheshi	15	05.6
	Dalit	12	04.4

Education status	Literate	189	70.0
	Illiterate	81	30.0

Occupation	Clerical, shop owner, farmer	89	33.0
	Unemployed	69	25.6
	Skilled worker	42	15.6
	Semiprofession	24	8.9
	Profession	23	8.5
	Unskilled worker	12	4.4
	Semiskilled worker	11	4.1

Poverty line	<1.9USD	61	22.6
	>1.9USD	209	77.4

Smoking tobacco products	Current	27	10.0
	Past	31	11.5
	Never	212	78.5

Alcohol consumption	Never	185	68.5
	Ex-alcoholic	25	09.3
	Regular	26	10.0
	Occasional	34	12.6

Waist circumference	Normal	149	55.2
	High	121	44.8

BMI	Underweight	4	1.5
	Normal	59	21.9
	Overweight	60	22.2
	Obese	147	54.4

BP	Yes	86	32
	No	184	68

Duration of diabetes	<5 years	142	52.6
	5‐10 years	91	33.7
	>10 years	37	13.7

Other illness	Yes	155	57.4
	No	115	42.6

Complication	Yes	51	18.9
	No	219	81.1

Diabetes in family	Yes	167	61.9
	No	103	38.1

Perception of disease	Serious	184	68.1
	Not serious	86	31.9

On medication^*∗*^	OHA	220	81.5
	Insulin	19.0	6.7
	Both	14.0	5.2

Hospital visit	Yes	253	93.7
	No	17	06.3

Private doctor visit	Yes	216	80.0
	No	54	20.0

ER visit	Yes	22	08.1
	No	248	91.9

Hospital admission	Yes	16	05.9
	No	254	94.1

Awareness about PHC	Yes	154	57
	No	116	43

Services available at PHC*∗*	OPD	152	99.3
	Immunization	133	86.9
	Laboratory	64	41.8
	Health education	26	17.0
	Specific for diabetes	19	12.4

Prefer PHC	Yes	5	3.3
	No	148	96.7

**Table 2:** Descriptive study of the D-39 questionnaire.

D-39 domains	No. of items	Mean	Median	SD	Minimum	Maximum
Energy and mobility	15	38.40	38.09	9.29	11.90	62.38
Diabetes control	12	48.59	48.80	8.60	17.86	67.86
Anxiety and worry	4	57.34	57.14	11.08	14.29	85.71
Social overload	5	39.75	38.57	10.14	12.86	64.29
Sexual behavior	3	29.29	30.95	13.44	7.14	64.29
Overall quality of life	39	42.95	42.45	8.11	15.93	64.28

**Table 3:** Association of demographic characteristics with QOL domains.

Characteristics	Energy and mobility mean ± SD	Diabetes control mean ± SD	Anxiety and worry mean ± SD	Social overload mean ± SD	Sexual behavior mean ± SD	Overall QOL mean ± SD
*Age in years*						
<50	33.40 ± 8.028	45.04 ± 8.08	54.04 ± 10.57	36.00 ± 10.20	26.91 ± 12.89	38.93 ± 7.20
>50	41.95 ± 8.49	51.10 ± 8.08	59.67 ± 10.87	42.42 ± 9.24	30.98 ± 13.61	45.80 ± 7.50
*p* value	<0.001	<0.001	<0.001	<0.001	0.014	<0.001

*Gender*						
Male	38.13 ± 9.79	48.54 ± 8.43	56.32 ± 11.37	40.58 ± 9.631	29.90 ± 13.83	42.88 ± 8.25
Female	38.68 ± 8.79	48.64 ± 8.81	58.36 ± 10.72	38.91 ± 10.61	28.67 ± 13.06	43.02 ± 7.98
*p* value	0.625	0.929	0.131	0.175	0.456	0.886

*Ethnicity*						
Janajati	36.45 ± 8.83	46.56 ± 8.68	55.52 ± 11.89	38.33 ± 9.90	29.19 ± 13.45	41.20 ± 8.01
Brahmin/Chhetri	39.50 ± 9.90	49.52 ± 8.67	58.36 ± 11.03	39.81 ± 10.35	29.18 ± 14.06	43.77 ± 8.43
Others	39.36 ± 7.92	50.15 ± 7.63	58.23 ± 9.06	42.54 ± 9.66	29.8 ± 111.84	44.29 ± 6.81
*p* value	0.038	0.015	0.140	0.068	0.960	0.030

*Marital status*						
Unmarried	30.76 ± 10.09	35.95 ± 9.93	47.14 ± 8.14	31.71 ± 7.45	19.52 ± 9.87	33.30 ± 8.78
Married	38.54 ± 9.24	48.83 ± 8.42	57.53 ± 11.05	39.90 ± 10.13	29.47 ± 13.45	43.13 ± 8.00
*p* value	0.063	0.001	0.038	0.074	0.101	0.007

*Education*						
Illiterate	41.04 ± 8.54	49.83 ± 8.12	58.55 ± 11.23	42.27 ± 9.83	34.36 ± 12.53	45.18 ± 7.77
Literate	37.27 ± 9.40	48.06 ± 8.77	56.82 ± 11.01	38.67 ± 10.10	27.12 ± 13.26	41.99 ± 8.08
*p* value	0.002	0.120	0.240	0.007	<0.001	0.003

*Occupation*						
Unemployed	42.22 ± 9.41	50.65 ± 9.78	60.24 ± 12.30	42.87 ± 9.68	30.26 ± 12.78	45.83 ± 8.80
Employed	37.09 ± 8.90	47.88 ± 8.07	56.34 ± 10.48	38.68 ± 10.10	28.96 ± 13.68	41.96 ± 7.63
*p* value	<0.001	0.037	0.020	0.003	0.489	0.001

*Poverty line*						
<1.9	37.93 ± 09.28	47.32 ± 09.50	57.14 ± 12.52	39.55 ± 10.16	28.84 ± 13.31	42.30 ± 08.47
>1.9	38.54 ± 09.31	48.96 ± 08.31	57.40 ± 10.66	39.81 ± 10.16	29.42 ± 13.51	43.14 ± 08.01
*p* value	0.651	0.192	0.874	0.860	0.767	0.476

*Current smoking status*						
Yes	37.23 ± 8.86	46.11 ± 7.94	53.17 ± 11.48	38.67 ± 8.44	27.60 ± 14.21	41.04 ± 7.00
No	38.53 ± 9.35	48.86 ± 8.65	57.80 ± 10.96	38.87 ± 10.32	29.48 ± 13.37	43.17 ± 8.20
*p* value	0.490	0.116	0.039	0.499	0.491	0.198

*Current alcohol (drinking) status*						
Yes	35.09 ± 8.10	46.11 ± 6.96	53.63 ± 10.51	36.42 ± 7.57	26.11 ± 12.62	39.86 ± 6.06
No	39.35 ± 9.41	49.30 ± 8.91	58.40 ± 11.04	40.70 ± 10.60	30.20 ± 13.56	43.83 ± 8.41
*p* value	0.002	0.004	0.003	0.001	0.037	<0.001

*Blood pressure*						
Normotensive	37.22 ± 9.38	47.71 ± 8.27	56.30 ± 10.67	38.90 ± 9.89	28.22 ± 13.24	45.10 ± 8.30
Hypertensive	40.89 ± 8.64	50.43 ± 9.04	59.52 ± 11.66	41.55 ± 10.50	31.55 ± 13.65	41.93 ± 7.83
*p* value	0.002	0.015	0.025	0.044	0.057	0.002

*BMI (kg/m* ^*2*^)						
<25	39.64 ± 9.67	49.42 ± 8.61	59.03 ± 11.66	40.33 ± 10.52	29.67 ± 13.90	43.96 ± 8.53
>25	37.37 ± 8.87	47.89 ± 8.56	55.92 ± 10.41	39.27 ± 9.82	28.97 ± 13.09	42.11 ± 7.66
*p* value	0.046	0.145	0.022	0.391	0.672	0.064

*Duration of diabetes (years)*						
<5	34.88 ± 8.10	46.06 ± 8.32	55.48 ± 9.78	37.18 ± 9.93	26.45 ± 12.91	40.08 ± 7.20
>5	42.21 ± 8.99	51.39 ± 8.05	59.40 ± 12.07	42.61 ± 9.63	32.44 ± 13.37	46.13 ± 7.90
*p* value	<0.001	<0.001	0.004	<0.001	<0.001	<0.001

*Comorbidity*						
Yes	41.65 ± 8.54	51.20 ± 8.17	59.49 ± 11.17	42.20 ± 9.95	32.05 ± 13.92	39.17 ± 7.09
No	34.02 ± 8.45	45.07 ± 7.92	54.44 ± 10.31	36.45 ± 9.48	25.56 ± 11.84	45.75 ± 7.68
*p* value	<0.001	<0.001	<0.001	<0.001	<0.001	<0.001

*Complication*						
Yes	45.44 ± 8.96	53.96 ± 7.64	63.16 ± 11.37	44.95 ± 8.70	31.60 ± 13.33	48.75 ± 7.58
No	36.77 ± 8.60	47.34 ± 8.35	55.98 ± 10.60	38.54 ± 10.09	28.75 ± 13.44	41.60 ± 7.63
*p* value	<0.001	<0.001	<0.001	<0.001	0.173	<0.001

*Family history of diabetes*						
Yes	39.09 ± 9.82	50.11 ± 8.48	59.30 ± 10.82	40.33 ± 10.06	28.78 ± 13.92	43.92 ± 8.34
No	37.29 ± 8.29	46.12 ± 8.26	54.16 ± 10.80	38.82 ± 10.24	30.12 ± 12.65	41.38 ± 7.50
*p* value	0.109	<0.001	<0.001	0.235	0.429	0.012
OHA	38.25 ± 8.68	48.82 ± 7.60	57.37 ± 10.13	39.68 ± 9.79	29.57 ± 13.28	42.11 ± 6.33
Insulin	45.63 ± 8.40	54.41 ± 6.989	63.86 ± 11.07	44.45 ± 8.65	26.19 ± 14.28	48.51 ± 6.50
Both	44.90 ± 8.79	55.52 ± 5.63	65.05 ± 9.62	47.14 ± 9.50	37.75 ± 10.00	47.44 ± 4.99
*p* value	<0.001	<0.001	0.002	0.005	0.442	0.025

*Hospital visit in last 6 months*						
Yes	38.72 ± 8.98	49.11 ± 7.85	57.72 ± 10.38	39.77 ± 10.05	29.39 ± 13.25	43.28 ± 7.60
No	33.70 ± 12.51	40.82 ± 14.33	51.68 ± 18.21	39.41 ± 11.77	27.8 ± 17.65	38.01 ± 12.90
*p* value	0.031	0.031	0.194	0.885	0.732	0.115

*Private doctor in last 6 months*						
Yes	38.46 ± 8.93	48.97 ± 7.94	57.45 ± 10.12	39.97 ± 9.92	28.83 ± 12.70	43.09 ± 7.52
No	38.18 ± 10.72	47.06 ± 10.81	56.88 ± 14.40	38.89 ± 11.02	31.12 ± 16.10	42.38 ± 10.20
*p* value	0.845	0.228	0.781	0.483	0.334	0.630

*ER visit in last 6 months*						
Yes	44.80 ± 8.51	54.76 ± 8.24	63.31 ± 11.36	45.84 ± 9.64	33.33 ± 13.60	49.01 ± 7.84
No	37.84 ± 9.17	48.04 ± 8.44	56.81 ± 10.92	39.21 ± 10.02	28.93 ± 13.40	42.41 ± 7.92
*p* value	0.001	<0.001	0.008	0.003	0.142	<0.001

*Hospital admission in last 6 months*						
Yes	43.87 ± 11.43	52.68 ± 11.96	60.50 ± 15.90	44.82 ± 11.53	34.52 ± 13.85	47.68 ± 11.20
No	38.06 ± 9.06	48.33 ± 8.31	57.14 ± 10.72	39.43 ± 9.99	28.96 ± 13.38	42.65 ± 7.80
*p* value	0.015	0.050	0.242	0.039	0.109	0.016

**Table 4:** Multiple linear regression analysis of significant factors associated with QOL and its domains.

QOL and its domain variables	Unstandardized coefficients	95% CI	*p* value
*Energy and mobility*			
Age	5.460	3.504–7.415	<0.001
Education	2.421	0.50–4.34	0.014
Current alcoholic	−2.78	−4.90−(−0.65)	0.011
Duration of diabetes in years	2.69	2.74–4.63	0.007
Comorbidity	2.58	0.57–4.60	0.012
Complication	5.937	3.62–8.25	<0.001
Hospital visit	4.70	1.143–8.25	0.010
ER visit	4.60	1.43–7.76	0.005
Constant	26.447		

*Diabetes control*			
Age	4.25	2.37–6.12	<0.001
Marital status	8.87	2.44–15.30	0.007
Current alcoholic	−2.60	−4.70–(−0.52)	0.014
Comorbidity	2.33	0.37–4.30	0.020
Complication	4.48	2.17–6.80	<0.001
Family history	1.60	−0.24–3.45	0.088
Hospital visit	6.96	3.36–10.55	<0.001
ER visit	4.36	1.20–7.52	0.007
Constant	27.919		

*Anxiety and worry*			
Age	4.24	1.76–6.71	0.001
Marital status	8.70	−0.37–(−17.74)	0.060
Current alcoholic	−3.65	−6.61–(−0.70)	0.016
BMI	−2.65	−5.12–(0.17)	0.036
Complication	5.18	2.01–8.35	0.001
Family history	3.66	1.11–6.21	0.005
ER visit	5.03	0.58–9.47	0.027
Constant	44.93		

*Social overload*			
Age	4.03	1.61–6.45	0.001
Education	2.86	0.43–5.30	0.021
Current alcoholic	−3.63	−5.33–(0.89)	0.006
Comorbidity	2.79	0.311–5.27	0.028
Complication	4.41	1.53–7.30	0.003
ER visit	5.64	1.66–9.63	0.006
Constant	35.84		

*Sexual behavior*			
Education	7.46	4.15–10.76	<0.001
Disease duration	2.86	−0.41–6.12	0.086
Comorbidity	5.65	2.37–8.92	0.001
Constant	24.276		

*Overall QOL*			
Age	3.97	2.21–5.72	<0.001
Marital status	7.02	1.22–12.82	0.018
Education	1.90	0.17–3.68	0.032
Current alcoholic	−2.115	−4.05–(-0.18)	0.032
BMI	−1.40	−3.03–0.23	0..092
Disease duration	1.84	0.09–3.60	0.039
Comorbidity	2.81	0.97–4.64	0.003
Complication	4.76	2.70–6.83	<0.001
ER visit	4.94	2.12–7.78	0.001
Constant	30.615		

## Data Availability

The data used to support the findings of this study are available from the corresponding author upon request.
